# Is the Right to Abortion at Risk in Times of COVID-19? The Italian State of Affairs within the European Context

**DOI:** 10.3390/medicina57060615

**Published:** 2021-06-12

**Authors:** Gianluca Montanari Vergallo, Raffaella Rinaldi, Valeria Piersanti, Anastasio Tini, Alessandro Del Rio

**Affiliations:** 1 Department of Anatomical, Histological, Forensic, and Orthopedic Sciences, Sapienza University of Rome, 00161 Rome, Italy; gianluca.montarivergallo@uniroma1.it (G.M.V.); raffa.rinaldi@uniroma1.it (R.R.); valeria.piersanti@uniroma1.it (V.P.); 2 Department of Excellence of Biomedical Sciences and Public Health, University “Politecnica delle Marche” of Ancona, 60020 Ancona, Italy; anastasio.tini78@gmail.com

**Keywords:** COVID-19, abortion, telemedicine, Italian legislation, European backdrop

## Abstract

The COVID-19 health emergency has thrown the health systems of most European countries into a deep crisis, forcing them to call off and postpone all interventions deemed not essential or life-saving in order to focus most resources on the treatment of COVID-19 patients. To facilitate women who are experiencing difficulties in terminating their pregnancies in Italy, the Ministry of Health has adapted to the regulations in force in most European countries and issued new guidelines that allow medical abortion up to 63 days, i.e., 9 weeks of gestational age, without mandatory hospitalization. This decision was met with some controversy, based on the assumption that the abortion pill could “incentivize” women to resort to abortion more easily. In fact, statistical data show that in countries that have been using medical abortion for some time, the number of abortions has not increased. The authors expect that even in Italy, as is the case in other European countries, the use of telemedicine is likely to gradually increase as a safe and valuable option in the third phase of the health emergency. The authors argue that there is a need to favor pharmacological abortion by setting up adequately equipped counseling centers, as is the case in other European countries, limiting hospitalization to only a few particularly complex cases.

## 1. Introduction: The COVID-19 Emergency and Termination of Pregnancy

The SARS-CoV-2 pandemic has sent shock waves all over the world, profoundly impacting every aspect of social life. In order to cope with the emergency, most European governments, including Italy’s, have had to reorganize health services and postpone all non-essential surgeries and procedures so as to prioritize care to COVID-19 patients [1]. Overwhelmed hospitals have also led to ethically controversial decisions as to what patients should be gaining access to care first [2]. Health systems all over the world have been challenged and disrupted by soaring demand for care of COVID-19 patients. To make matters worse, factors such as fear, stigma, misinformation, and limitations on movement have bogged down the delivery of health care for a wide array of life-threatening conditions, such as cancer and cardiovascular diseases [3,4,5]. In fact, when health care systems are overwhelmed, patients may not be able to access necessary care, hence direct mortality from COVID-19 outbreaks and indirect mortality from preventable and treatable conditions both increase dramatically. What can really make a difference is trust from the general public in the capabilities of health care systems to meet essential needs in a safe and effective fashion, while keeping in check the infection risk in health facilities [6]. The pandemic has just as profoundly affected public health. As several reports have pointed out, COVID-19 and the resulting economic recession have negatively affected many people’s mental health. Concerns about mental health and substance use have been growing [7]. High levels of deteriorating mental conditions, substance use, and suicidal ideation were reported by many different studies in several countries [8,9,10]. In addition, people already afflicted by mental illness and substance use disorders [11] have had to face new obstacles and challenges threatening their prospects of recovery [12,13,14]. As a matter of fact, failing to consistently provide care for mental, neurological, and substance disorders can be life-threatening, such as in the cases of discontinuation of care for epilepsy, unavailable harm reduction services, unaddressed suicide risk, and unmanaged severe drug and alcohol withdrawal syndromes [15,16,17]. The pandemic is also jeopardizing the nutritional well-being of vulnerable populations through multiple unfolding dynamics and mechanisms [18]. Dietary quality and quantity are expected to fall due to the loss of household income and disruptions in food systems (e.g., disruption of trade and transport of foods from production to markets) and school feeding programs. In addition, the uncharted territory in which health care professionals have been operating has led many to argue that some form of medical liability protection [19,20] ought to be put in place, in order to shield doctors from possible lawsuits due to decisions made under unprecedented circumstances, with no protocols to guide them [21]. Within such an extremely troublesome context, women who want to terminate a pregnancy have had difficulty in finding operational counseling institutions and hospital services [22,23], mostly due to the redistribution of resources and personnel, in addition to travel limitations.

In Europe, abortion is one of the most common procedures for women of reproductive age, with an annual incidence rate ranging from 6.4 per 1000 women aged 15 to 44 (Switzerland) to 19.2 per 1000 (Sweden). It is a safe and well-established procedure [24]. Still, if practiced in unsafe conditions, it can have consequences on a woman’s health. A delay in the execution can bring about irreparable damage both for those women forced to undergo surgery at a more advanced stage of pregnancy, because the risk of death increases by 30% for each week of gestation, and for women who can no longer terminate their pregnancies because they have gone over the legal time frame [25,26]. On the one hand, the pandemic and the ensuing economic hardships have increased the likelihood of unwanted pregnancies, along with other factors, such as higher rates of sexual violence [27] and limited access to contraception. In an effort to “bend the curve” of the pandemic, European governments, including the Italian one, have suspended health services for abortion or have reassigned gynecological ward staff to COVID-19 units. Additionally, the necessary information on abortion services available during the pandemic has not been provided to patients. Those developments have forced many women to go abroad and to face the difficulties arising from bans and restrictions to counter the pandemic, and the sense of worry and distress progressively compounds as time goes by [28]. This situation is particularly worrisome for women who live in the few European states where abortion is illegal or severely restricted, such as in Malta [29], and who cannot travel abroad to seek assistance and treatment. MSI Reproductive Choices has estimated that an additional 2.7 million abortions will occur outside the formal health setting (i.e., self-managed abortions [30]), while the World Health Organization warns that reducing the availability of essential services of Sexual and Reproductive Health and Maternal and Neonatal Health will result in thousands of maternal and neonatal deaths, due to “millions of unintentional pregnancies and unsafe abortions [6]”. According to data provided by the Ministry of Health, updated to 10 March 2021, the total number of total COVID-19 cases exceeds three million (3,123,368) and over 100,000 (100,811) fatalities have been registered. Current public health measures to mitigate and control the pandemic are focused on the use of masks and personal protective devices, social distancing, and frequent sanitation of environments and facilities. This approach does entail several issues in terms of stemming the spread of the disease. It is hoped that large-scale vaccination campaigns will decisively limit the viral spread and shorten the duration of the pandemic and its impact in terms of morbidity and mortality.

With the struggle against the pandemic still far from over, the Council of Europe [31] called on all member states to ensure full access to reproductive health, to urgently remove all obstacles preventing access to safe abortions and uphold the right of all to rely on the highest standard of health, including sexual and reproductive health [32]. Therefore, since access to abortion is necessary to preserve the health and safety of many women, all related services should have continued to be guaranteed with all the essential safety measures in place to limit the spread of the infection without infringing upon the freedom of choice and the right to health [33]. Regrettably, that was not the case.

## 2. Voluntary Termination of Pregnancy in Italy

Italy was one of the countries most intensely affected by the burgeoning pandemic, and earlier than most. The health emergency from COVID-19 has highlighted the shortcomings of the health system, unprepared to face a massive demand for services. Territorial medicine based on family doctors was unable to stem the infections in a timely fashion, and COVID-19 patients were forced to turn to hospitals which, in turn, were unable to cope with the strong pressure, due to the constant decrease in economic resources which health care staff and the number of beds suffered over the last few decades [34]. For such reasons, access to voluntary termination of pregnancy, already usually problematic due to the high number of conscientious objectors among health professionals, has become even harder to obtain. Conscientious objection, acknowledged as a personal right and codified in several codes of medical ethics, among which is the Italian one under Article 22, is valid only if declared in advance and provided that it does not jeopardize the patient’s health [35]. Conscience-based refusal still poses major issues, however. Data for 2018 show that 69% of gynecologists, 46.3% of anesthesiologists, and 42.2% of non-medical personnel are conscientious objectors, with regional variations [36]. It is for this very reason that the European Committee of Social Rights (CEDS) has twice [37] condemned Italy over the violation of the right to health of women, including its 2016 decision decrying the discrimination suffered by non-objecting health care personnel [38]. The committee has in fact ascertained that many hospitals could not provide abortion services, and that patients in some cases had to move to other regions, or even go abroad due to extremely long waiting lists [39]. The Committee has therefore urged the Italian government to ensure a more homogeneous distribution of non-objecting personnel, and the effectiveness of the service throughout the national territory within October 2019 [40]. The inability to terminate one’s pregnancy in due time also negatively affects the right enshrined in law no. 194 of 22 May 1978, since pregnancy in Italy can only be legally terminated within specific time limits.

### 2.1. Law n.194/1978 and Its Core Provisions

Voluntary termination of pregnancy in Italy is governed by law no.194/1978 and can take place within the first ninety days (Articles 4 and 5) and after the first ninety days (Articles 6 and 7) [41]. As is known, the woman can legally have an abortion within the ninetieth gestational day if she believes that the continuation of her pregnancy could entail “a serious danger to her physical or mental health, in relation to her state of health, or to her economic, social or family conditions, or the circumstances in which conception took place, or expects fetal anomalies or malformations” (Art. 4, co. 1) [42]. After this term, the conditions for requesting an abortion are stricter, and are related to cases in which pregnancy or childbirth involves a serious danger to the life of the woman, or there are significant fetal anomalies that endanger physical or mental health (Article 6, co. l). Furthermore, in order to undergo termination of pregnancy within the first trimester, the woman needs to turn to a family clinic (or a trusted doctor) where, at the end of an interview focused on the reasons that lead her to make this choice, the doctor issues a document, countersigned by the woman, which certifies the state of the pregnancy along with the request [43] for termination. At that point, the woman needs to wait seven days and only afterwards can she undergo surgery at a health facility authorized to carry out terminations of pregnancy (Art. 5, par. 3). The obstacles caused by the COVID-19 pandemic added to this already burdensome picture for women in 2020.

### 2.2. Voluntary Termination of Pregnancy through Pharmacological Methods in Italy: The Time Limit and the Hospitalization Mandate in the 2010 Guidelines

The COVID-19 emergency has brought to the fore the issue of the increased demand for medical procedures to terminate pregnancy instead of surgical ones [44]. The voluntary termination of pregnancy through pharmacological methods is based on the administration of two different agents, mifepristone (better known as RU486) and a prostaglandin, within 48 h of each other [45]. Although emergency contraception is ethically controversial, with some health care operators refusing to prescribe or provide it on grounds of conscience [46], the WHO has included the two abortion drugs in the list of essential medicines [47]. Mifepristone is a synthetic anti-progestogen that affects the progesterone receptors, which are necessary for maintaining pregnancy. Associated with a prostaglandin, it makes the embryo no longer viable, thus terminating the pregnancy [48]. It is taken orally, does not require surgery and anesthesia and, from a clinical standpoint, does not require hospitalization, although the whole process usually takes three days, since 48 h have to elapse between taking mifepristone and prostaglandin. It can be used in the first weeks of pregnancy, while aspiration is generally performed after the 7th week. In Italy this method has been in use since 2009 (in France since 1988 and in the UK since 1990). When these drugs were first marketed, the Italian Medicines Agency (AIFA) authorized their administration by the forty-ninth day of amenorrhea, with mandatory hospitalization in one of the facilities authorized to carry out abortions, until the expulsion of the product of conception. The Agency has also specified that “the entire abortion process must take place under the supervision of a doctor of the obstetric and gynecological service who also had the task of correctly informing the patient about the use of the medicine, the drugs to be combined, the alternative methods and possible related risks, as well as constantly monitoring the patient in order to minimize the occurrence of reported adverse reactions, such as haemorrhages, infections and fatal events” [49]. Following the AIFA determination and the related opinion of the Italian High Council of Health [50], the Ministry of Health issued a set of guidelines for medical abortion which prescribed the intake of the two drugs within the seventh week of pregnancy in ordinary hospitalization [51]. Such a method, however, is not evenly applied nationwide. In fact, it ranges between 44% of the total number of abortions performed in Piedmont and 1–2% in the Autonomous Province of Bolzano and the southern region of Molise. In addition, a significant gap exists in the use of such drugs between the Northern Regions and the others [52]. It is worth noting that the data reported in the Ministry Report show that the pharmacological method is safe and effective overall, considering that in 96.9% of cases the intake of the two drugs did not give rise to any complications, and only in 5.3% of cases was it necessary to resort to hysterosuction or interventions of the uterine cavity to complete the procedure. The situation further improved in 2018, with only 2.4% of cases needing to resort to hysterosuction or revision of the uterine cavity to complete the operation. In 88.5% of terminations, hospitalization was shorter than 24 h, and in 4.8% of cases the woman was hospitalized for one night only. In 2018, there were 15,750 voluntary abortions with RU486, 20.8% of the total, a strong increase compared to the first years of use, when the rate was well below 10%. In fact, since 2005 some Regions (Lombardy, Tuscany, Emilia-Romagna, Latium, and Umbria until June 2020), based on Art. 15 of the law, which provides for the updating of health personnel on the use of the most advanced techniques more respectful of the physical and mental integrity of the woman and less risky for the termination of pregnancy, have ordered the administration of the drug [53] in a three-day hospitalization regime, considering it a more appropriate path “in light of the clinical assistance assessments of professionals based on data from international literature”. Some regions, including Umbria, had already departed from the ministerial guidelines since 2005 and had introduced the one-day hospitalization regime, by virtue of the safety of the method [54]. In the regions that applied the hospitalization regime, 76% of women had circumvented the requirement by choosing voluntary discharge after taking the first drug and booking a new hospitalization after two days to complete the procedure. The choice of voluntary discharge was strongly discouraged by doctors because the abortion could take place outside the hospital and involve serious risks for the woman’s health

### 2.3. The Ministry of Health Directive Issued on 12 August 2020: “Update of the Guidelines on Voluntary Termination of Pregnancy with Mifepristone and Prostaglandins”

The reorganization of health care facilities caused by the pandemic has led some freedom of choice advocacy groups [55] to request greater use of pharmacological abortion to protect women’s health and their rights, jeopardized by the ongoing health care emergency. The traditional procedure of surgical abortion calls for numerous outpatient examinations, for the certification and dating of the pregnancy, and for preoperative examinations, as well as hospital admittance for the actual procedure. Therefore, it exposes women to an excessive number of contacts within health structures, which are certainly not conducive to the need to reduce contacts which may increase the risk of contagion. It is evident that a more extensive use of pharmacological abortion, hitherto limited to a rather marginal role, would make it possible to ease the pressure on hospitals, anesthesiologists, and surgical wards [56]. In light of such considerations, on 12 August 2020 the Italian Ministry of Health ordered the updating of the guidelines issued in 2010; specifically, the new guidelines authorize “the use of voluntary termination of pregnancy with a pharmacological methods for up to 63 days, i.e., 9 completed weeks of gestational age and its administration in public adequately equipped outpatient facilities, connected to hospitals and licensed by Regional authorities, as well as counseling centers, or day hospitals, based on the already mentioned recommendation by the World Health Organization, and following the favorable opinion of the Superior Health Council of 4 August 2020” [28]. The new guidelines cancel the requirement of hospitalization for the drug RU486 and extend the time frame of its use by two weeks [57]. At the same time, the Italian Medicines Agency removed the requirement for the drug Mifegyne to be used in hospitalization only, and extended its use up to the 63rd day of gestational age in counseling centers or day hospitals [58]. The issuance of new guidelines, which we feel are more in keeping with scientific evidence and medical best practices, should be viewed as a positive development. Firstly, since the greater difficulties that women encounter in accessing voluntary abortion services risk exceeding the time limits set by Law 194/78, an even greater risk is posed for women who live in conditions of marginalization and vulnerability, in precarious health conditions or positivity to COVID-19 [59]. Instead, pharmacological abortion with the two-week extension can provide an effective solution both for women and for easing the pressure on overwhelmed hospitals, reducing the inflow and average hospitalization time of patients, a meaningful contribution in times of unprecedented emergency. The two-week extension also has a significant impact on time allocation, because it enables healthcare staff to follow multiple patients at the same time, reduces the use of surgical procedures, and allows healthcare facilities to carry out, if clinical circumstances permit and the woman agrees, less demanding services in terms of economic and human resources, with a substantially positive impact on health care provision and management overall. The new guidelines, as devised by the legislature, should fulfill a connecting function, in that they incentivize the regional governments to put in place homogeneous and common procedures, while preserving the degree of organizational autonomy to which they are entitled. In reality, however, that did not come to fruition. The update carried out by the Italian Health Care Ministry was positively received by those who for years had advocated for the modification of the 2010 guidelines—even more so in view of the new pandemic recrudescence that loomed in early 2021, although it was criticized by those who believed that ordinary hospitalization should always be required in all circumstances [60]. Several regions have not yet enforced the updated guidelines, some regions such as Umbria have even refused to abide by them [61], and in many clinics the RU486 is not yet administered due to several “bureaucratic–administrative” issues [62].

## 3. Pharmacological Abortion and the Potential of Telemedicine

The World Health Organization has acknowledged the potential benefits of telemedicine in terms of guaranteeing an acceptable level of health care services [6,63]. Throughout the daunting health emergency brought about by COVID-19, with mandatory social distancing, reduced mobility, and hospital closures, telemedicine through video calls proved to be valuable, because it enabled patients to not go to the hospital, drastically reducing the risk of infection both for patients and healthcare professionals, in addition to ensuring truly patient-centered care.

In countries such as Sweden and Denmark, telemedicine has already been well-established and widespread for years [64], while other countries such as England, Wales, Scotland, and France used it during the first wave of the COVID-19 pandemic, in order to cope with the difficulties caused by the growing health emergency. Almost all countries have also used it for medical abortions, which allowed patients to stay home and to rely on the virtual assistance of their doctors. The results were ultimately positive and encouraging, with medical abortions exceeding half of the total at the end of June 2020, i.e., a few months after the appearance of COVID 19, reducing the need to move and the consequent risk to spread the infection [65]. Furthermore, the degree of efficacy and safety of telemedicine is similar to that reported with “in-person” care, with a post-procedure surgical revision rate ranging from 0.9 to 19.3% [66]. Remote assistance in Italy has also become part of the National Health Service in 2020 [67]. Among the services provided, there are virtual consultation, counseling, assistance, and reporting, but not yet voluntary interruption of pregnancy, despite the fact that the Italian Society of Gynecology and Obstetrics (SIGO) has recommended laying out a set of standards for medical abortion through a “totally remote procedure monitored by telemedicine services” [68]. Hence, authorizing and implementing medical abortion provided through telemedicine services, in addition to being a safe and effective option [69], is even more necessary in the third phase of the health emergency. In fact, it would help to solve many issues related to the persistent discontinuation or reduction in services in some hospitals, as was already the case during the first wave of the pandemic, and would guarantee the right to health and self-determination of women.

## 4. Coronavirus and Voluntary Termination of Pregnancy: The Policies Implemented by European States

During the COVID-19 pandemic, European governments have unevenly regulated access to abortion services. Some countries (Andorra, Liechtenstein, Malta, Monaco, San Marino) continued to ban it for non-medical reasons. In Poland, a Constitutional Court ruling issued on 22 October 2020 allowed the termination of pregnancy only in cases of high probability of irreversible or fatal damage to the fetus, or if the mother’s life is in danger, and in cases of incest and rape [70,71]. Northern Ireland legalized voluntary termination of pregnancy for any reason up to the 12th week of pregnancy with the 2019 Northern Ireland Act. It extended the limit to the 24th week in cases where the continuation of pregnancy would entail a risk to the physical or mental health of the mother or if the fetus is suffering from a severe physical or mental handicap. Medical abortion was authorized on 9th April 2020 through misoprostol home use [72]. Some countries, including Hungary, restrict access to abortion because they consider it a non-essential procedure [73]. Other countries, such as the Netherlands, Belgium, Germany, Iceland, Latvia, Luxembourg, Montenegro, Slovenia, England, Wales, and Scotland, deny access to abortion for women who have tested positive for COVID-19, who must wait for the symptoms to disappear in order to be admitted to abortion. The World Health Organization has also recommended the use of this method for up to the ninth gestational week [74]. In Europe, medical abortion is the most widespread procedure, carried out through outpatient programs or at home and can also be administered by family doctors (in France) and specifically trained midwives (in France and Sweden) [75]. Overall, most European countries have made few major changes to increase access to medical abortion. The limit within which it can be legally carried out is 9 weeks. In countries such as England, women will be able to take both pills for early medical abortion within 10 weeks in their own homes, without having to go to a hospital or clinic first [76,77]. Other European countries (Belgium, Estonia, Ireland, Finland, France, Germany, Norway, Portugal, Switzerland, England, Wales, Scotland, Northern Ireland, and Denmark) use remote technology to assist women during medical abortion at home. In some countries (Estonia, Belgium, Denmark, Northern Ireland, or Portugal) only misoprostol can be taken at home while mifepristone still has to be taken in a clinical setting. Abortion drugs can be delivered by mail in England, Wales, Scotland, and Georgia or delivered to patient homes in England, Wales, Scotland, and Ireland. Some nations have issued specific directives, meant to stay in force for the emergency period only. Germany has allowed mandatory pre-abortion counseling to take place by telephone or video-consultation rather than in person [61]. Portugal has waived the usually required waiting period and allowed postponement or follow-up via telemedicine, but in-person visits are still required for the provision of the service [78]. Ultimately, it is safe to conclude that during the pandemic, European governments have failed to come up with a uniform response to ensure continuity and reliability of care for women seeking to terminate their pregnancies. The national responses ranged from the choice to further restrict access to abortion (Poland, Hungary) to progressive actions that expanded up to 10 weeks the time frame in which medical abortion can be legally provided and supported the use of telemedicine to that end.

## 5. A Few Closing Remarks

Abortion “is an essential service of global health care”, the lack of which “profoundly affects a person’s life, health and well-being” [79,80].

In the midst of the pandemic, along with the general reorganization of health care facilities, many hospitals have reduced or suspended access to voluntary termination of pregnancy in order to reassign premises and personnel for the care of COVID-19 patients. Consequently, many women have struggled to find clinics and hospital services to terminate their pregnancies, and have not even been able to travel abroad, as the pandemic has made traveling much harder, as a result of grounded airports and border closures to stem the viral spread. By the approval of the new guidelines on pharmacological abortion, the Italian legislator has sought to ensure that the right to abortion can be upheld throughout the country, as requested by the European Committee of Social Rights. The issuance of the new guidelines, however, has raised concerns and controversy. Those who are opposed to medical abortion have expressed the fear that the woman could end up without the necessary level of assistance. In reality, pharmacological abortion is a health treatment. Women must therefore be informed by their doctors about effectiveness, methods of execution, indications and contraindications in order to make an informed decision possible. Essentially, the Italian law has structured the doctor–patient relationship on the basis of the so-called therapeutic alliance model. According to such a model, doctors are required to inform their patients in an understandable and thorough fashion “about the diagnosis, prognosis, benefits and risks of the diagnostic tests and health treatments indicated, as well as the possible alternatives and the consequences of any refusal” (as codified in Article 1, paragraph 3), while it is up to the patient’s decision-making autonomy to choose whether to start or continue a given treatment or assessment, as well as to choose which treatment or assessment to follow between those alternatively presented whenever possible. The principle of informed consent constitutes an expression of autonomy or, better still, a means for the fulfillment of the self-determination principle in healthcare. Even the Constitutional Court in its ruling no. 438 of 2008 [81] clarified that the right to abortion no longer refers exclusively to the right to health—as it was when law no. 194 of 1978 was enacted—but also an expression of the woman’s self-determination on her own body through her procreative choices. The path of medical abortion in Italy unequivocally shows that it was precisely the self-determination of women that guided the legislature’s choices towards the pharmacological option, since the monitoring of the implementation of law no. 194 found that more than three quarters of women who undergo the medical procedure choose voluntary discharge, thus exercising their right to treatment and informed consent. Statistical data bear out the advantages of medical abortion: in countries where a high percentage of women choose the abortion pill—such as in France or Germany—the number of surgical abortions has not increased at all. Furthermore, facilitating medical abortion does not mean trivializing abortion—which will be a sensitive and often extremely painful choice—but rather it enables those who have already decided to terminate their pregnancy to figure out the best ways to do it. In our opinion, especially in times of pandemics, it is necessary to promote the local governance of pharmacological abortion, with well-equipped counseling centers and specifically trained personnel, so as to limit hospitalization to only the rare, more problematic cases, as has been the case for some time in most European countries with very high rates of medical abortion. At the same time, the optimization and harmonization of shared European abortion oversight systems would be advisable, in order to make sure that women who choose to terminate their pregnancies can be supported in a safe and timely fashion, and to even out the existing discrepancies in Europe in terms of abortion accessibility and waiting periods. Ultimately, improving support and information-sharing networks is of utmost importance: counselling and practical support for women need to be ensured, particularly when the main reason why a woman seeks to have an abortion is rooted in family or economic pressure, as highlighted in a Council of Europe resolution [82].

## Data Availability

The data presented in this study are available on request from the corresponding author.
